# DNA Barcode Gap Analysis for Multiple Marker Genes for Phytoplankton Species Biodiversity in Mediterranean Aquatic Ecosystems

**DOI:** 10.3390/biology11091277

**Published:** 2022-08-27

**Authors:** Eftychia Tzafesta, Benedetta Saccomanno, Francesco Zangaro, Maria Rosaria Vadrucci, Valeria Specchia, Maurizio Pinna

**Affiliations:** 1 Department of Biological and Environmental Sciences and Technologies, DiSTeBA, University of Salento, via Monteroni 165, 73100 Lecce, Italy; 2 Research Centre for Fisheries and Aquaculture of Acquatina di Frigole, DiSTeBA, University of Salento, 73100 Lecce, Italy; 3 Regional Agency for the Environmental Prevention and Protection (ARPA Puglia), Corso Trieste 27, 70126 Bari, Italy

**Keywords:** phytoplankton, aquatic ecosystems, eDNA metabarcoding and eDNA, marker genes, genetic distances

## Abstract

**Simple Summary:**

Environmental DNA metabarcoding (eDNA) has strong potential in the assessment of biodiversity in aquatic ecosystems. The incompleteness of DNA barcode reference libraries represents a current limit to unveiling the whole biodiversity of an aquatic ecosystem. Therefore, barcode gap analyses at species level are of great significance, in particular at local/regional level, for the advancement of eDNA metabarcoding application to aquatic ecosystems surveillance and future biodiversity assessment.

**Abstract:**

The implementation of DNA metabarcoding and environmental DNA (eDNA) to the biodiversity assessment and biomonitoring of aquatic ecosystems has great potential worldwide. However, DNA metabarcoding and eDNA are highly reliant on the coverage of the DNA barcode reference libraries that are currently hindered by the substantial lack of reference sequences. The main objective of this study was to analyze the current coverage of DNA barcode reference libraries for phytoplankton species of the aquatic Mediterranean ecoregion in the southeast of Italy (Apulia Region) in order to assess the applicability of DNA metabarcoding and eDNA in this area. To do so, we investigated three main DNA barcode reference libraries, BOLD Systems, GenBank and SILVA, for the availability of DNA barcodes of the examined phytoplankton species. The gap analysis was conducted for three molecular gene markers, 18S, 16S and COI. The results showed a considerable lack of barcodes for all three markers. However, among the three markers, 18S had a greater coverage in the reference libraries. For the 18S gene marker, the barcode coverage gap across the three types of ecosystems examined was 32.21–39.68%, 60.12–65.19% for the 16S marker gene, and 72.44–80.61 for the COI marker gene. Afterwards, the interspecific genetic distance examined on the most represented molecular marker, 18S, was able to distinguish 80% of the species mined for lakes and 70% for both marine and transitional waters. Conclusively, this work highlights the importance of filling the gaps in the reference libraries, and constitutes the basis towards the advancement of DNA metabarcoding and eDNA application for biodiversity assessment and biomonitoring.

## 1. Introduction

Biodiversity assessments are fundamental for ecological management and conservation, specifically for aquatic ecosystems. Biomonitoring, as well as the analysis of abundance and occurrence of target taxonomic groups and species, is essential for evaluating the ecological health status of marine coastal, transitional, and freshwater ecosystems and their environmental changes [1,2,3]. Phytoplankton has been extensively used as one of the biological quality elements in different aquatic ecosystems, due to their rapid response to environmental variations and anthropogenic pressures [4,5]. Furthermore, phytoplankton communities have an essential role in food webs and biogeochemical cycles, and consequently in the ecological functioning of the aquatic ecosystems [4,6]. Recently phytoplankton biodiversity is emerging as an important factor for ecosystems’ functioning in changing climatic conditions [7]. Phytoplankton is a multi-taxa group, including unicellular and colonial organisms of different sizes, shapes, types of metabolism, and life cycles. Phytoplankton assemblages express high diversity, and this characteristic influences the resilience and the efficient use of ecosystems resources [8,9].

Phytoplankton organisms are commonly identified by light or electronic microscopy-based techniques [10]. Microscope based methods involve the identification of phytoplankton species based on morphological and other visible criteria. Currently, the estimation of the phytoplankton biodiversity depends on the skills and experience of operators to correctly distinguish distinctive taxonomic species traits.

In recent years, genetic diversity assessment is becoming a useful tool for phytoplankton species identification. DNA-based identification of species refers to the sequencing of a partial target gene, and the alignment of the obtained sequence with reference sequences, called DNA barcodes, deposited in specific reference libraries. The public and most commonly used DNA barcode reference libraries are GenBank by the National Center for Biotechnology Information (NCBI) [11], the Barcode of Life Data System (BOLD Systems) [12] and SILVA database [13]. The DNA barcoding target genes are evolutionary conserved and present a sufficient difference in the nucleotide sequence to differentiate species from different taxa. They include COI, commonly used for animal barcoding, rRNA genes, such as 12S, 18S and 16S that are used for phytoplankton and bacteria, the nuclear ribosomal internal transcribed spacer 1 and 2 (ITS) for fungi, and the large subunit of ribulose 1,5-bisphosphate carboxylase-coding gene (rbc-L) for plants [14,15,16].

High-throughput sequencing technologies allow for the sequencing of the target gene of different species present in an environmental sample [17]. Several studies have used eDNA metabarcoding for phytoplankton biodiversity assessment in different aquatic ecosystems [18,19].

However, a current a limit of the metabarcoding analyses is the incompleteness of the DNA barcode reference libraries. The reference libraries need to be as comprehensive and curated as possible, in order to facilitate the assignment of sequences to species. Their deficiency has been investigated for different aquatic taxonomic groups and geographic regions. Significant absence of DNA barcodes was quantified for macroinvertebrate species [20,21,22], for ascidians and cnidarians [23], for some major invertebrate macrofauna phyla [24], and recently for freshwater fish, aquatic insects and molluscs [25]. Yet, to our knowledge, there is only one study that investigated the DNA barcodes availability for phytoplankton species and it revealed a considerably low coverage [20]. This study investigates the current status of incompleteness of DNA barcode reference libraries for phytoplankton species of aquatic ecosystems from the Mediterranean ecoregion, specifically in the southeast of Italy (Apulia Region). The study also analyzes the sequence polymorphism for DNA-based species delimitation of phytoplankton with publicly available 18S, 16S and COI records. This study is preliminary and important for the effective application of DNA-based biodiversity assessments in this specific ecoregion.

## 2. Materials and Methods

### 2.1. Phytoplankton Checklist and Gap-Analysis

For the present study, a comprehensive species checklist of the Italian Apulia Regional Environmental Protection Agency (ARPA-Puglia, Regional report 2011) was acquired, consisting of phytoplankton species from the most significant aquatic ecosystems of the Apulia Region in southeast Italy (Mediterranean ecoregion). The phytoplankton species were separated into three lists according to the ecosystem they were found: transitional waters, lakes, and marine coastal waters. Species names were verified using the worldwide platform WORMS (http://www.marinespecies.org; last access on 12 November 2021).

The listed species were analyzed for the presence or absence of DNA barcodes for the three gene markers, 18S, 16S and COI, in the three DNA barcode libraries, BOLD Systems; GenBank and SILVA (last access on 12 November 2021). It should be mentioned that the SILVA library contains only 18S and 16S sequences and information. The percentage of each phylum and family with or/and without barcodes in the reference libraries was assessed. Furthermore, in order to verify if the analysis of multiple gene markers improves the number of identified species and the potential DNA metabarcoding applications, we analyzed the overlap of species with DNA barcode for the three gene markers for each ecosystem.

### 2.2. Species Delimitation Analysis

All publicly available 18S, COI and 16S sequences of taxa with species-specific names were downloaded from NCBI and BOLD using PrimerMiner 0.3b. If no records were found, ribosomal sequences were manually downloaded from SILVA. For each of them, we aligned the sequences of all the accessions to generate one consensus sequence.

These consensus sequences were then aligned with ClustalW in MEGAX and their ends were trimmed to obtain the same length across all the input sequences, while those with insufficient overlap were discarded. 18S sequences resulted in a fragment of about 300 bp (base pairs) in position 1000–1300 for lake species, in position 600–900 for marine coastal species, and in position 700–1000 for transitional waters species. COI sequences resulted in a fragment of 230 bp in position 230–460 for marine coastal species, and 350 bp in position 300–650 bp for transitional waters, while lake species were not analyzed because only six species in the dataset had a COI barcode. 16S sequences resulted in a fragment of about 350 bp in position 300–670 for lake species, and in positions 500–850 for marine coastal and transitional waters species.

These alignments were used to construct a maximum likelihood phylogenetic tree with rapid bootstrap (100 replicates) using the general time reversible + gamma (GTR+G) model in RAxml (Randomized Axelerated Maximum Likelihood) version 8.2.12. The resulting tree was used to infer species delimitation with the Bayesian implementation of the Poisson Tree Processes (bPTP) model with 100,000 MCMC generation and 1% burn-in and then annotated with FigTree v1.4.4 (last access on May 2022).

## 3. Results

### 3.1. Gap Analysis of Phytoplankton Species

**Figure 1:**
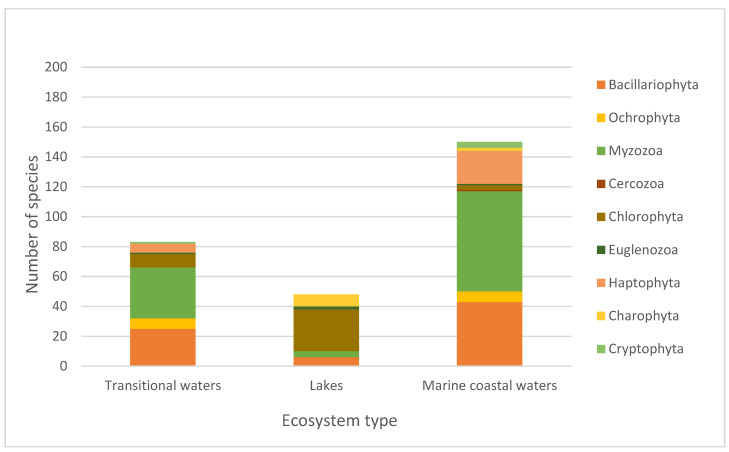
Number of phytoplankton species without an 18S barcode for the different phyla in transitional waters, lakes, and marine coastal waters.

**Figure 2:**
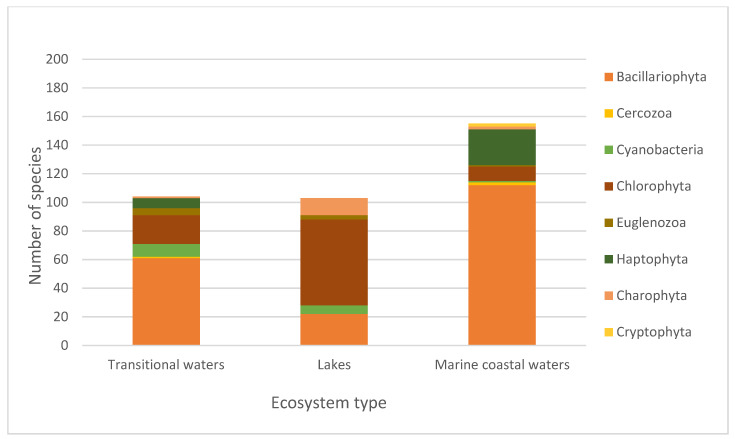
Number of species without a 16S barcode for the different phyla in transitional waters, lakes, and marine coastal waters.

**Figure 3:**
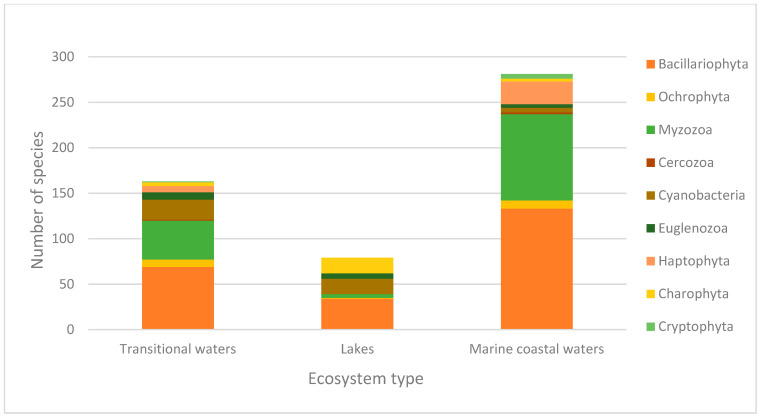
Number of species without a COI barcode for the different phyla in transitional waters, lakes, and marine coastal waters.

In order to analyse the incompleteness of the DNA barcode sequences in the reference libraries that are useful for biodiversity assessment and applications of eDNA metabarcoding for biomonitoring, we used the list of phytoplankton species from the aquatic ecosystems identified in the three categories described above. The list comprised 250 species from transitional waters, 169 species from lakes, and 383 species from marine coastal waters. These phytoplankton species belonged to 10 phyla, 19 classes and 88 families in transitional waters, 8 phyla, 17 classes and 58 families in lakes, and 10 phyla, 22 classes and 94 families in marine coastal waters. The listed species were analyzed for the presence or absence of DNA barcodes for the three genetic markers (18S, 16S and COI) in the barcode libraries (BOLD Systems, GenBank and SILVA) (Supplementary Tables S1–S9). For the 18S rRNA gene marker, the current total lack of barcodes is 36.73% for species from transitional waters, 32.21% for species from lakes, and 39.68% for species from marine coastal waters. For the 16S rRNA gene marker, the current total DNA barcode coverage is significantly lower than the 18S rRNA coverage, with a 60.12% of lack of barcodes for species from transitional waters, 65.19% for species from lakes, and 64.32% for species from marine coastal waters. The COI gene marker has the lowest coverage among all the markers, with a remarkable lack of barcodes for species from lakes (80.61%), 72.44% for species from transitional waters, and 76.57% for species from marine coastal waters (Table 1). In particular, the distribution of the species without DNA barcodes among the different phylum for every aquatic ecosystem category and marker is shown in the Figure 1, Figure 2 and Figure 3 (see also Supplementary Tables S1–S9).

**Figure 4:**
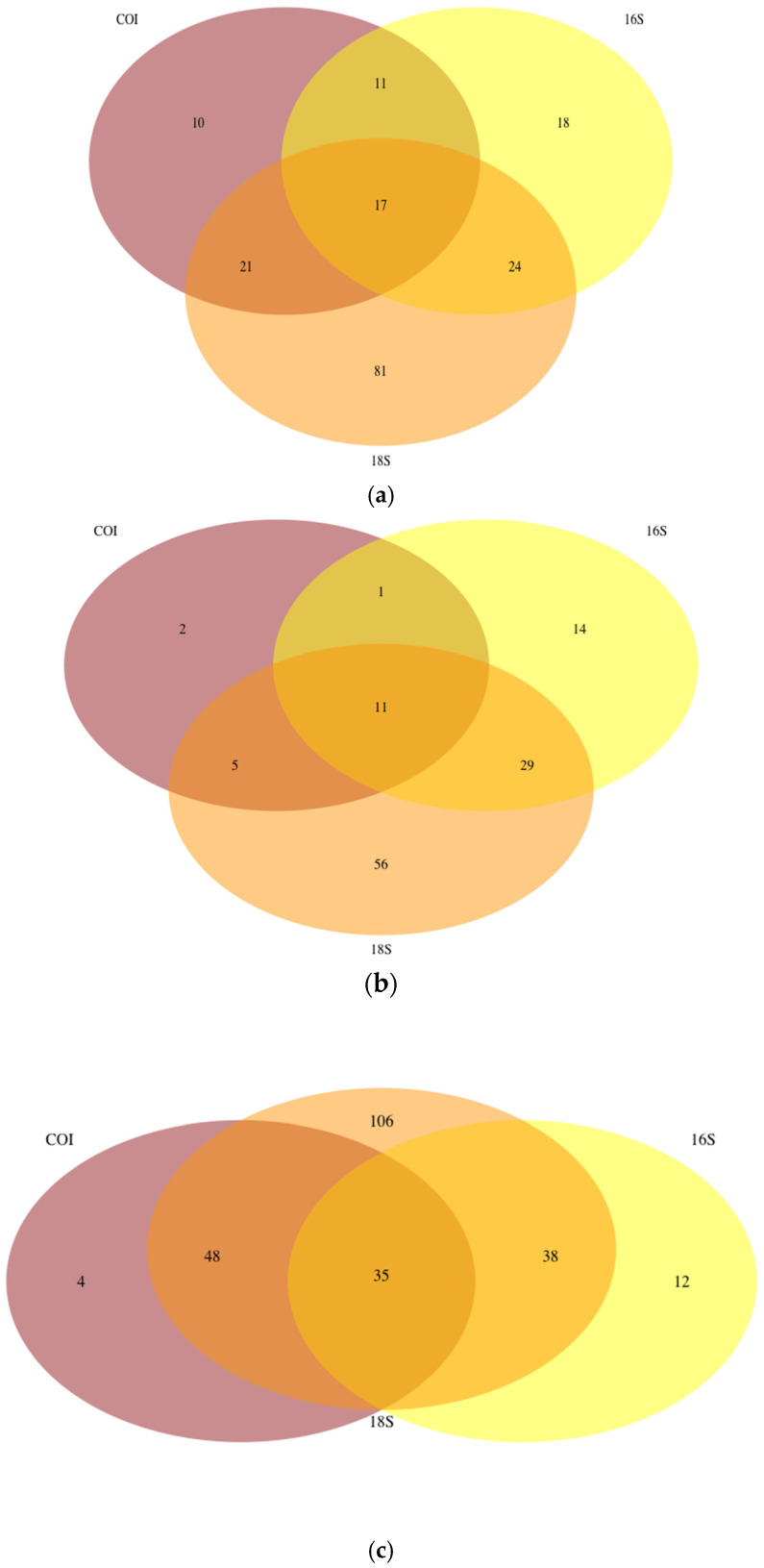
Overlap of species with DNA barcode for the three markers (COI, 16S, and 18S) from transitional waters (**a**), lakes (**b**), and marine coastal waters (**c**).

An overlap analysis of the species with DNA barcode for the three gene markers was completed to estimate whether the use of multiple markers could improve the application of eDNA metabarcoding. The 18S gene marker had the greatest coverage in the reference libraries of the three studied markers for all three ecosystem types. However, the overlap analysis of the species with DNA barcode for the three gene markers showed that the use of multiple gene markers could improve the species’ identification efficiency, at least for transitional water ecosystems (Figure 4).

### 3.2. Interspecific Genetic Distance Analysis

The efficiency in species identification could be influenced from the inter-specific genetic distances. We analyzed the genetic distances among species belonging to the ecosystems types for the three barcodes.

**Figure 5:**
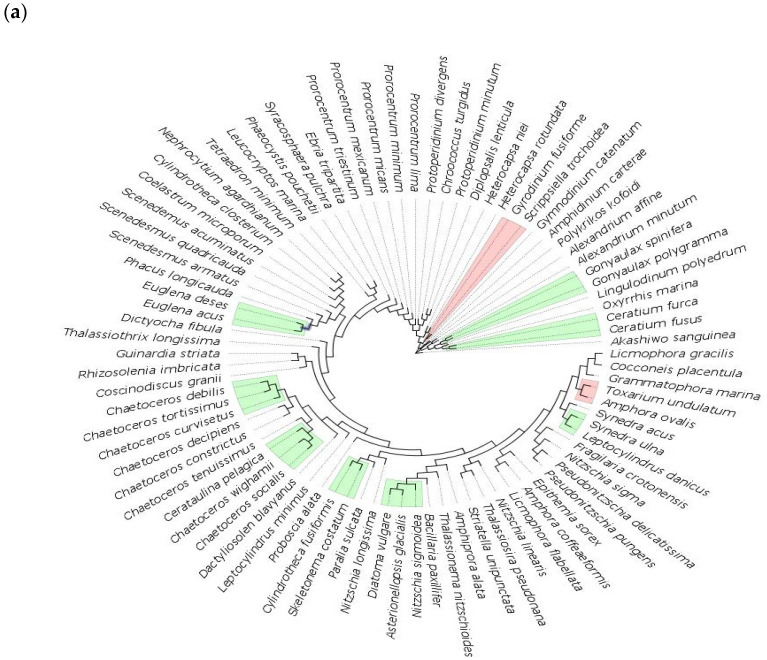

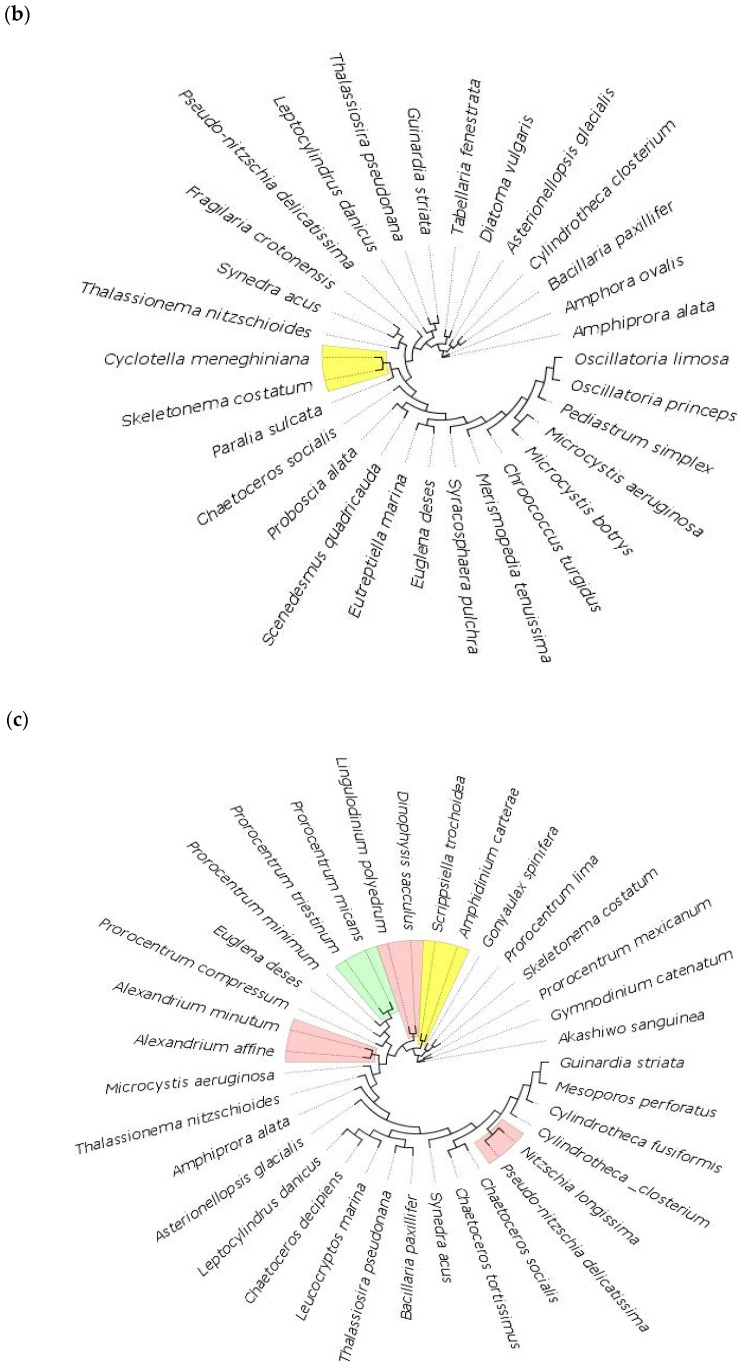
Maximum likelihood phylogenetic tree (GTR+I+G4 model of substitution with 100 bootstrap replicates) of transitional water species for 18S marker (**a**), 16S marker (**b**) and COI marker (**c**). Colored branches indicate species that cluster together under bPTP analysis, with green representing Bayesian support (BS) values of 0.3 or below, yellow for BS 0.3–0.5, red for BS 0.5–0.6.

In transitional waters, the number of putative species delimited bPTP analysis is 63 of 86 species (73%) with 18S, 28 of 30 (93%) with 16S, and 25 of 35 (71%) with COI. Among the delimited species, 26 (30%) with 18S, 9 (30%) with 16S, and 10 (29%) with COI exhibit Bayesian support values of 0.7 or above (Figure 5, Table S10).

The genus *Prorocentrum* is separated with the highest Bayesian values with 18S, while *P.micans* and *P.triestinum* cluster together with COI (0.218) and both *P.lima* and *P.mexicanum* have a BS value of only 0.3. In this dataset, the genus *Alexandrium* gets separated with 18S, but not with COI (0.5), as does *Nitzschia longissimi*, which is a single species in 18S (0.7) but is clustered with *Pseudonitzschia delicatissima* in COI. On the other hand, *Cylindrotheca fusiformis* and *Cylindrotheca closterium* are separated with COI (0.99), but not with 18S, where *C.fusiformis* is linked to *Skeletonema costatum* (0.24). The genus *Chaetoceros*, mostly represented by 18S sequences, did not separate well with only 3 out of 10 species resulting as a single species. However, the *Chaetoceros* spp. that clustered together all had BS values below 0.3, and two of these species could be distinguished by COI with a BS value of 0.5.

**Figure 6:**
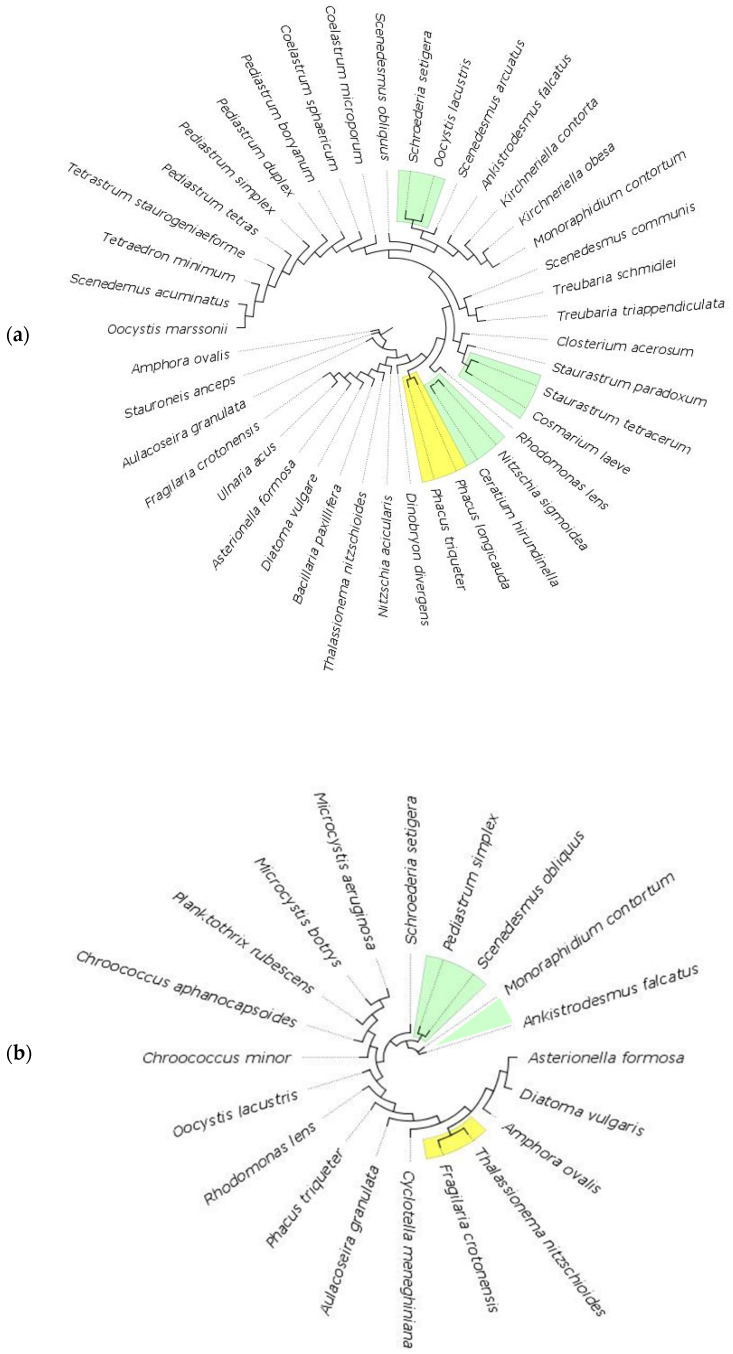
Maximum likelihood phylogenetic tree (GTR+I+G4 model of substitution with 100 bootstrap replicates) of lake species for 18S markers (**a**) and 16S marker (**b**). Colored branches indicate species that cluster together under bPTP analysis, with green representing Bayesian support (BS) values of 0.3 or below, yellow for BS 0.3–0.5, red for BS 0.5–0.6.

In lakes, the number of putative species delimited by bPTP analysis for 18S marker is a total of 33 out of 41 species (80%), of which 37% exhibit Bayesian support (BS) values above 0.7. Considering the species belonging to the same genus, four species of the *Pediastrum* genus and two of *Coelastrum* are separated with the highest Bayesian value, followed by the species in *Scenedesmus*, *Kirchneriella* and *Treubaria* genera. On the other hand, the two *Phacus* species clustered together with a BS value of 0.471 (Figure 6a, Table S11). Considering the 16S marker for prokaryotes and photoautotrophic eukaryotes, bPTP analysis was able to distinguish 14 out of 20 species (70%), of which 45% displayed a BS value above 0.7. Compared to 18S, it found higher BS values for *Amphora ovalis*, *Asterionella Formosa*, *Aulacoseira granulata*, *Diatoma vulgaris*, and *Rhodomonas lens*, however, it clusters *Thalassionema nitzschioides* with *Fragilaria crotonensis* (BS = 0.482), *Moraphindium contortum* with *Ankistrodesmus falcatus* (BS = 0.282) and *Pediastrum simplex* with *Scenedesmus obliquus* (BS = 0.282) (Figure 6b, Table S2). We could not make a comparison with the COI marker because only six of the species under study presented a COI barcode.

**Figure 7:**
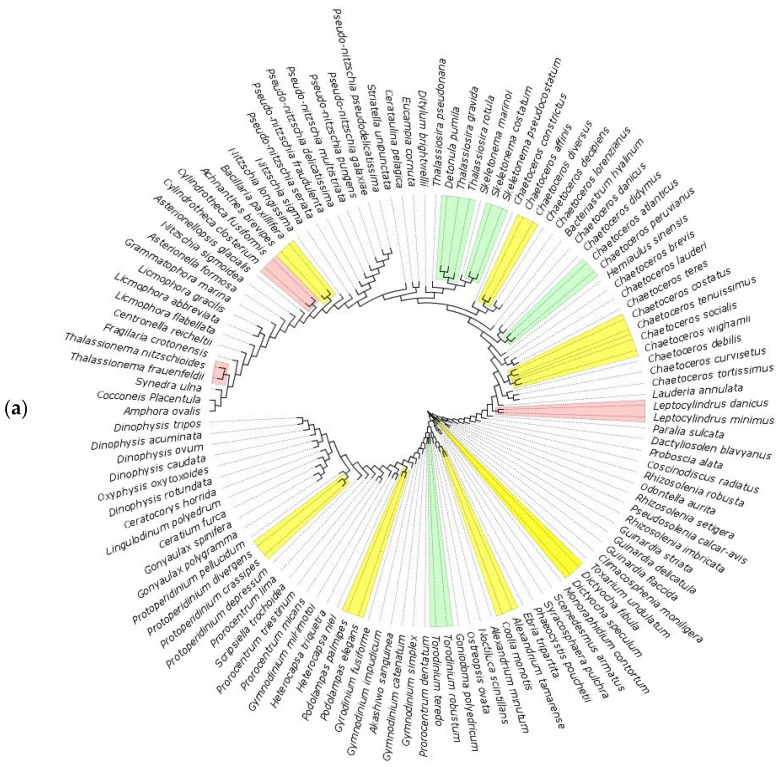

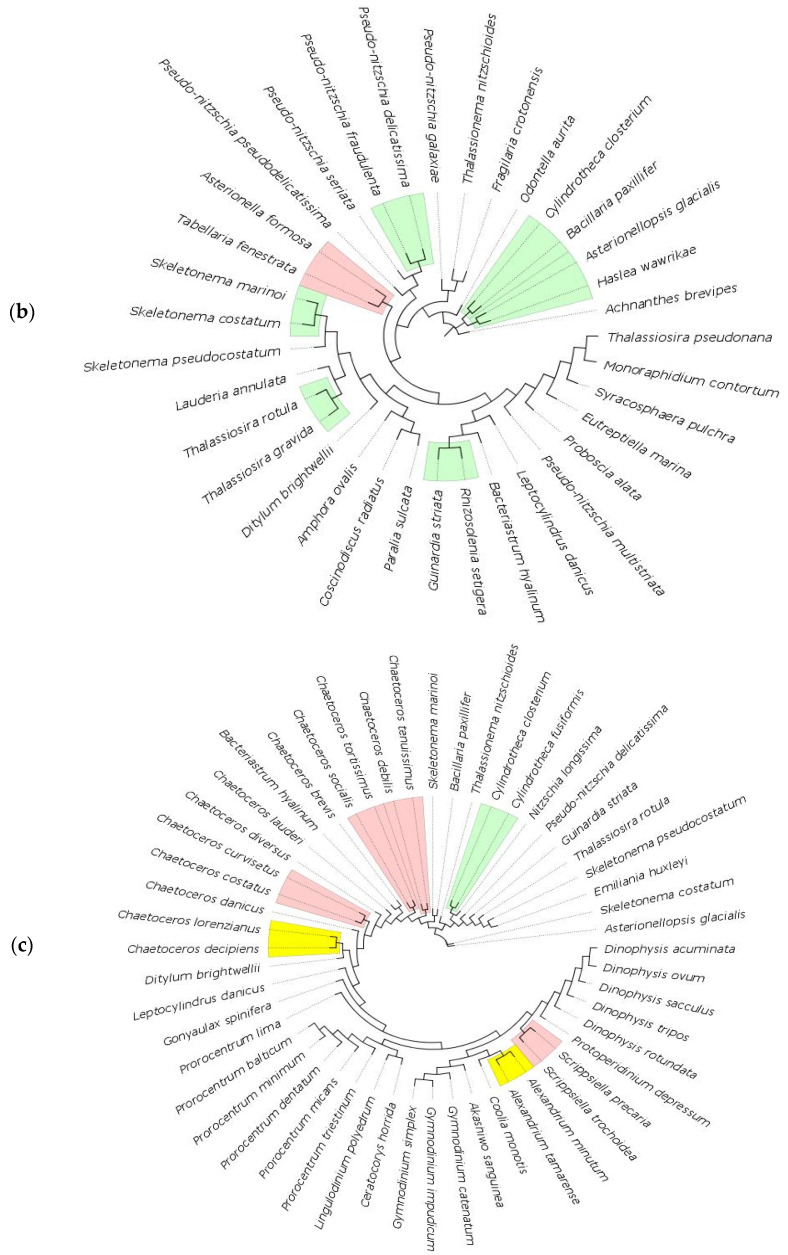
Maximum likelihood phylogenetic tree (GTR+I+G4 model of substitution with 100 bootstrap replicates) of marine coastal species for 18S markers (**a**), 16S marker (**b**) and COI marker (**c**). Colored branches indicate species that cluster together under bPTP analysis, with green representing Bayesian support (BS) values of 0.3 or below, yellow for BS 0.3–0.5, red for BS 0.5–0.6.

In marine coastal waters, the number of putative species delimited by bPTP analysis is a total of 86 of 121 species (71%) with 18S, 19 of 35 with 16S (54%) and 38 of 52 (73%) with COI. Among the delimited species, 48 (40%) with 18S, 10 (28%) with 16S, and 16 (42%) with COI exhibit Bayesian support values of 0.7 or above. Analysis with 18S is able to distinguish species of the genus *Gymnodium, Guinardia, Heterocapsa, Nitzschia, Pseudonitzschia* and *Rhizosolenia,* while the genus *Dinophysis, Prorocentrum* and *Skeletonema* are better separated with COI. On the other hand, 18S presents a better resolution than COI for the genera *Alexandrium* and *Chaetoceros*. The two species of the genus *Cylindrotheca* clustered together with both 18S and COI but the latter showed lower BS value (0.26 compared to 0.5). For 16S, where *C. fusiformis* is not present, *Cylindrotheca closterium* clustered together with *Bacillaria paxillifer* (BS = 0.251), which in turn clustered with *Achnanthes brevipes* in COI. Instead, *Achnanthes brevipes* is considered a single species with 16S (BS = 0.65) and Bacillaria paxillifer is considered a single species with COI (BS = 0.5) (Figure 7, Table S12).

## 4. Discussion and Conclusions

DNA metabarcoding has been recently advanced and recognized as an efficient way for evaluating biodiversity. In this study, we analyzed three major DNA barcode reference libraries (BOLD Systems, GenBank and SILVA) for the availability of DNA barcodes for the phytoplankton taxa of the aquatic ecosystems of the Apulia region in the southeast of Italy. The gap-analysis was conducted for three molecular markers; 18S, 16S and COI. We observed that a large percentage of the examined phytoplankton taxa lack a DNA barcode in the reference libraries. Our analysis showed that there is an urgent need to complete the reference libraries, which would facilitate the efficient use of DNA metabarcoding for biomonitoring.

In accordance with our observations, there are several other studies pointing out considerable gaps in the reference libraries for different taxa [20,21,22,23,24,25,26,27,28,29]. However, there are not many gap-analysis studies focusing on phytoplankton taxa. Recently, Weigand et al. [20] examined, among others, a list of diatom species from several European countries, including Italy, for the availability of rbcL and 18S molecular markers in the Diat.barcode library. Regarding Italy, the coverage was 37% when both markers were present, and 55% when at least one marker was present.

In metabarcoding studies, the use of multiple gene targets has been acknowledged for providing a wider taxonomic recovery [30]. For instance, an eDNA metabarcoding study of water samples from riverine and lake habitats in China amplified both 16S and 18S, aiming to investigate the biodiversity and community compositions of phytoplankton [31]. Accordingly, eDNA metabarcoding of COI and 18S markers was performed to survey eukaryotic biodiversity in water samples from a coral reef tract. Most of the taxa detected with 18S were phytoplankton, while COI mainly detected arthropods. There was an overlap of 33 genera (14%) of the taxa recovered with the two different markers, underlining the complementarity of these genes [32]. In our work, the advantage of multi-locus barcoding was particularly evident for the phytoplankton taxa of the transitional waters (Figure 4). We chose to focus on 18S, 16S and COI because, for now, they are recognized as the most universal markers across different taxa. However, when metabarcoding is only targeting phytoplankton species, it is impossible to explore additional markers like ribulose 1,5-biphosphate carboxylase (rbcL), which is known to be effective for the identification of primary producers.

In this study, we also wanted to show how molecular diversity could predict species delimitation solely based on genetic distances. The Bayesian implementation of the PTP model is often used in metabarcoding and metagenomic studies to infer inter and intra-specific differences and, thus, identify known species or delimit cryptic ones. Here, we used the model solely to demonstrate the importance of having up-to-date barcode libraries for the identification of species. Molecular species delimitation was able to distinguish 80% of the species mined for lakes and 70% for both marine and transitional waters. While 18S on its own has a good resolution, we demonstrated how the use of additional markers could clarify or confirm species delimitation. Besides, this analysis also indirectly highlights the difference in barcode coverage, with 18S sequenced in more than twice as many species as the other two markers. This is also why care should be taken in making comparisons among markers, as differences in the number of input species influence the bPTP output. Besides, it is important to outline that the sequences used in this analysis were consensus sequences created from all the publicly available accessions of each species. This means that a different number of accessions was used for each species, explaining why the Bayesian support values are often low even with a heterogeneous dataset. Nonetheless, the use of a small fragment of about 200–350 bp, even in these mock datasets, achieved good species delimitation if we consider that most species that clustered together had a BS value of 0.4 or below. We also recommend sequencing larger fragments in order to find as much overlap as possible with publicly available sequences. For example, we could not include many *Chaetoceros* in the analysis of COI for transitional water compared to marine coastal because the COI fragment of these species is upstream of the region used for the analysis of this dataset (350 bp in position 300–650 bp).

Molecular species delimitation techniques are informative for species-level sorting, but an essential requisite is the development and validation of molecular markers that can provide a reliable and robust taxonomic assignment. In particular, we recommend the development of an array of markers targeting several genes/barcodes, in order to overcome possible taxonomic misplacement due to a lack of dissimilarities based on only one single sequence.

## Figures and Tables

**Table 1:** Percentage of phytoplankton species without DNA barcode sequences in the reference libraries for each gene marker considered and aquatic ecosystem category.

	*Total 18S Barcode Gap*	*Total 16S Barcode Gap*	*Total COI Barcode Gap*
*Transitional waters*	36.73%	60.12%	72.44%
*Lakes*	32.21%	65.19%	80.61%
*Marine coastal waters*	39.68%	64.32%	76.57%

## Data Availability

Not applicable.

## Supplementary Materials

The following supporting information can be downloaded at: https://www.mdpi.com/article/10.3390/biology11091277/s1, Table S1: Number of species with and without COI barcode for the families from transitional waters; Table S2: Number of species with and without 16S barcode for the families from transitional waters; Table S3: Number of species with and without 18S barcode for the families from transitional waters; Table S4: Number of species with and without COI barcode for the families from lakes; Table S5: Number of species with and without 16S barcode for the families from lakes; Table S6: Number of species with and without 18S barcode for the families from lakes; Table S7: Number of species with and without COI barcode for the families from marine coastal waters; Table S8: Number of species with and without 16S barcode for the families from marine coastal waters; Table S9: Number of species with and without 18S barcode for the families from marine coastal waters; Table S10: Delimitation analysis results of transitional water species for markers 18S,16S and COI.; Table S11: Delimitation analysis results of lake species for markers 18s and 16s. Table S12: Delimitation analysis results of coastal-marine species for markers 18s,16s and COI.

## References

[1] Tilman D., Isbell F., Cowles J.M. (2014). Biodiversity and ecosystem functioning. Annu. Rev. Ecol. Evol. Syst..

[2] Pinna M., Marini G., Rosati I., Neto J.M., Patrício J., Marques J.C., Basset A. (2013). The usefulness of large body-size macroinvertebrates in the rapid ecological assessment of Mediterranean lagoons. Ecol. Indic..

[3] Van der Plas F. (2019). Biodiversity and ecosystem functioning in naturally assembled communities. Biol. Rev..

[4] Ward B.A., Dutkiewicz S., Jahn O., Follows M.J. (2012). A size-structured food-web model for the global ocean. Limnol. Oceanogr..

[5] Wu N., Schmalz B., Fohrer N. (2014). Study progress in riverine phytoplankton and its use as bio-indicator—A review. Austin J. Hydrol..

[6] Litchman E., Klausmeier C.A. (2008). Trait-based community ecology of phytoplankton. Annu. Rev. Ecol. Evol. Syst..

[7] Bestion E., Haegeman B., Codesal S.A., Garreau A., Huet M., Barton S., Montoya J.M. (2021). Phytoplankton biodiversity is more important for ecosystem functioning in highly variable thermal environments. Proc. Natl. Acad. Sci. USA.

[8] Gunderson L.H. (2000). Ecological resilience—in theory and application. Annu. Rev. Ecol. Evol. Syst..

[9] Ptacnik R., Solimini A.G., Andersen T., Tamminen T., Brettum P., Lepistö L., Willén E., Rekolainen S. (2008). Diversity predicts stability and resource use efficiency in natural phytoplankton communities. Proc. Natl. Acad. Sci. USA.

[10] Karlson B., Cusack C., Bresnan E., Intergovernmental Oceanographic Commission of UNESCO (2010). Microscopic and Molecular Methods for Quantitative Phytoplankton Analysis.

[11] Benson D.A., Cavanaugh M., Clark K., Karsch-Mizrachi I., Lipman D.J., Ostell J., Sayers E.W. (2012). GenBank. Nucleic Acids Res..

[12] Ratnasingham S., Hebert P.D. (2007). BOLD: The Barcode of Life Data System. Mol. Ecol. Notes.

[13] Quast C., Pruesse E., Yilmaz P., Gerken J., Schweer T., Yarza P., Peplies J., Glöckner F.O. (2012). The SILVA ribosomal RNA gene database project: Improved data processing and web-based tools. Nucleic Acids Res..

[14] Acinas S.G., Marcelino L.A., Klepac-Ceraj V., Polz M.F. (2004). Divergence and redundancy of 16S rRNA sequences in genomes with multiple rrn operons. J. Bacteriol..

[15] Badotti F., de Oliveira F.S., Garcia C.F., Vaz A.B.M., Fonseca P.L.C., Nahum L.A., Oliveira G., Góes-Neto A. (2017). Effectiveness of ITS and sub-regions as DNA barcode markers for the identification of Basidiomycota (Fungi). BMC Microbiol..

[16] CBOL Plant Working Group (2009). A DNA barcode for land plants. Proc. Natl. Acad. Sci. USA.

[17] Pawlowski J., Kelly-Quinn M., Altermatt F., Apothéloz-Perret-Gentil L., Beja P., Boggero A., Borja A., Bouchez A., Cordier T., Domaizon I. (2018). The future of biotic indices in the ecogenomic era: Integrating (e)DNA metabarcoding in biological assessment of aquatic ecosystems. Sci. Total Environ..

[18] Stoeck T., Kochems R., Forster D., Lejzerowicz F., Pawlowski J. (2018). Metabarcoding of benthic ciliate communities shows high potential for environmental monitoring in salmon aquaculture. Ecol. Indic..

[19] Apothéloz-Perret-Gentil L., Bouchez A., Cordier T., Cordonier A., Guéguen J., Rimet F., Vasselon V., Pawlowski J. (2021). Monitoring the ecological status of rivers with diatom eDNA metabarcoding: A comparison of taxonomic markers and analytical approaches for the inference of a molecular diatom index. Mol. Ecol..

[20] Weigand H., Beermann A.J., Čiampor F., Costa F.O., Csabai Z., Duarte S., Geiger M.F., Grabowski M., Rimet F., Rulik B. (2019). DNA barcode reference libraries for the monitoring of aquatic biota in Europe: Gap-analysis and recommendations for future work. Sci. Total Environ..

[21] Specchia V., Tzafesta E., Marini G., Scarcella S., D’Attis S., Pinna M. (2020). Gap analysis for DNA barcode reference libraries for aquatic macroinvertebrate species in the Apulia Region (Southeast of Italy). J. Mar. Sci. Eng..

[22] Vieira P.E., Lavrador A.S., Parente M.I., Parretti P., Costa A.C., Costa F.O., Duarte S. (2021). Gaps in DNA sequence libraries for Macaronesian marine macroinvertebrates imply decades till completion and robust monitoring. Divers. Distrib..

[23] Paz G., Rinkevich B. (2021). Gap analysis of DNA barcoding in ERMS reference libraries for ascidians and cnidarians. Environ. Sci. Eur..

[24] Hestetun J.T., Bye-Ingebrigtsen E., Nilsson R.H., Glover A.G., Johansen P.O., Dahlgren T.G. (2020). Significant taxon sampling gaps in DNA databases limit the operational use of marine macrofauna metabarcoding. Mar. Biodivers..

[25] Li F., Zhang Y., Altermatt F., Zhang X., Cai Y., Yang Z. (2022). Gap analysis for DNA-based biomonitoring of aquatic ecosystems in China. Ecol. Indic..

[26] Zangaro F., Saccomanno B., Tzafesta E., Bozzeda F., Specchia V., Pinna M. (2021). Current limitations and future prospects of detection and biomonitoring of NIS in the Mediterranean Sea through environmental DNA. NeoBiota.

[27] Pinna M., Saccomanno B., Marini G., Zangaro F., Kabayeva A., Khalaj M., Shaimardan L., D’Attis S., Tzafesta E., Specchia V. (2021). Testing the Influence of Incomplete DNA Barcode Libraries on Ecological Status Assessment of Mediterranean Transitional Waters. Biology.

[28] Jażdżewska A.M., Tandberg A.H.S., Horton T., Brix S. (2021). Global gap-analysis of amphipod barcode library. PeerJ.

[29] Leite B.R., Vieira P.E., Teixeira M.A., Lobo-Arteaga J., Hollatz C., Borges L.M.S., Duarte S., Troncoso J.S., Costa F.O. (2020). Gap-analysis and annotated reference library for supporting macroinvertebrate metabarcoding in Atlantic Iberia. Reg. Stud. Mar. Sci..

[30] Deagle B.E., Jarman S.N., Coissac E., Pompanon F., Taberlet P. (2014). DNA metabarcoding and the cytochrome c oxidase subunit I marker: Not a perfect match. Biol. Lett..

[31] Zhang L., Yang J., Zhang Y., Shi J., Yu H., Zhang X. (2022). eDNA biomonitoring revealed the ecological effects of water diversion projects between Yangtze River and Tai Lake. Water Res..

[32] Sawaya N.A., Djurhuus A., Closek C.J., Hepner M., Olesin E., Visser L., Kelble C., Hubbard K., Breitbart M. (2019). Assessing eukaryotic biodiversity in the Florida Keys National Marine Sanctuary through environmental DNA metabarcoding. Ecol. Evol..

