# Supplementary material for: DNA Barcode Gap Analysis for Multiple Marker Genes for Phytoplankton Species Biodiversity in Mediterranean Aquatic Ecosystems
Source: Biology (Basel). 2022 Aug 27;11(9):1277. doi: 10.3390/biology11091277 (PMC9495960; doi:10.3390/biology11091277)
Supplement: Supplementary file 1 [file biology-11-01277-s001.zip › Supplementary_tables_S1-S9.pdf]

Table S1. Number of species with and without COI barcode for the families from transitional waters

Phylum	Family	Total Number of species	Number of species without barcode	Number of species with barcode	Percentage of gap in the libraries
Bacillariophyta	*Achnanthaceae*	1	1	0	100,00
Bacillariophyta	*Cocconeidaceae*	4	4	0	100,00
Bacillariophyta	*Fragilariaceae*	8	5	3	62,50
Bacillariophyta	*Chaetocerotaceae*	12	5	7	41,67
Bacillariophyta	*Climacospheniaceae*	1	1	0	100,00
Bacillariophyta	*Coscinodiscaceae*	3	2	1	66,67
Bacillariophyta	*Stephanodiscaceae*	3	2	1	66,67
Bacillariophyta	*Bacillariaceae*	12	5	7	41,67
Bacillariophyta	*Diploneidaceae*	3	3	0	100,00
Bacillariophyta	*Pinnulariaceae*	1	0	1	0,00
Bacillariophyta	*Stauroneidaceae*	1	1	0	100,00
Bacillariophyta	*Amphipleuraceae*	2	1	1	50,00
Bacillariophyta	*Naviculaceae*	3	2	1	66,67
Bacillariophyta	*Pleurosigmataceae*	3	3	0	100,00
Bacillariophyta	*Hemiaulaceae*	2	2	0	100,00
Bacillariophyta	*Leptocylindraceae*	2	1	1	50,00
Bacillariophyta	*Cymbellaceae*	1	1	0	100,00
Bacillariophyta	*Gomphonemataceae*	1	1	0	100,00
Bacillariophyta	*Rhizosoleniaceae*	7	6	1	85,71
Bacillariophyta	*Paraliaceae*	1	1	0	100,00
Bacillariophyta	*Striatellaceae*	3	2	1	66,67
Bacillariophyta	*Rhopalodiaceae*	1	1	0	100,00
Bacillariophyta	*Melosiraceae*	2	2	0	100,00
Bacillariophyta	*Catenulaceae*	3	3	0	100,00
Bacillariophyta	*Skeletonemaceae*	4	2	2	50,00
Bacillariophyta	*Thalassiosiraceae*	1	0	1	0,00
Bacillariophyta	*Surirellaceae*	1	1	0	100,00
Bacillariophyta	*Licmophoraceae*	3	3	0	100,00
Bacillariophyta	*Tabellariaceae*	1	1	0	100,00
Bacillariophyta	*Thalassionemataceae*	6	5	1	83,33
Bacillariophyta	*Mastogloiaceae*	1	1	0	100,00
Bacillariophyta	*Toxariaceae*	1	1	0	100,00
Ochrophyta	*Chrysococcaceae*	1	1	0	100,00
Ochrophyta	*Dinobryaceae*	4	2	2	50,00
Ochrophyta	*Dictyochacea*	2	2	0	100,00
Ochrophyta	*Pleurochloridaceae*	2	2	0	100,00
Ochrophyta	*Pedinellaceae*	1	1	0	100,00
Myzozoa	*Gymnodiniaceae*	6	3	3	50,00
Myzozoa	*Polykrikaceae*	2	2	0	100,00
Myzozoa	*Gyrodiniaceae*	3	3	0	100,00
Myzozoa	*Amphidiniaceae*	4	3	1	75,00

Myzozoa	*Dinophysaceae*	1	0	1	0,00
Myzozoa	*Peridiniaceae*	4	1	3	25,00
Myzozoa	*Oxytoxaceae*	2	2	0	100,00
Myzozoa	*Heterocapsaceae*	4	4	0	100,00
Myzozoa	*Protoperidiniaceae*	13	13	0	100,00
Myzozoa	*Gonyaulacaceae*	6	2	4	33,33
Myzozoa	*Ceratiaceae*	2	2	0	100,00
Myzozoa	*Ostreopsidaceae*	3	0	3	0,00
Myzozoa	*Tovelliaceae*	1	1	0	100,00
Myzozoa	*Thoracosphaeraceae*	1	0	1	0,00
Myzozoa	*Oxyrrhinaceae*	1	1	0	100,00
Myzozoa	*Prorocentraceae*	14	6	8	42,86
Cercozoa	*Ebriaceae*	1	1	0	100,00
Cyanobacteria	*Nostocaceae*	1	0	1	0,00
Cyanobacteria	*Chroococcaceae*	5	5	0	100,00
Cyanobacteria	*Microcystaceae*	4	3	1	75,00
Cyanobacteria	*Gomphosphaeriaceae*	1	1	0	100,00
Cyanobacteria	*Oscillatoriaceae*	5	5	0	100,00
Cyanobacteria	*Coelosphaeriaceae*	3	3	0	100,00
Cyanobacteria	*Merismopediaceae*	3	3	0	100,00
Cyanobacteria	*Spirulinaceae*	2	2	0	100,00
Euglenozoa	*Astasiaceae*	1	1	0	100,00
Euglenozoa	*Eutreptiaceae*	3	2	1	66,67
Euglenozoa	*Phacidae*	2	2	0	100,00
Euglenozoa	*Euglenidae*	1	1	0	100,00
Euglenozoa	*Euglenaceae*	3	2	1	66,67
Haptophyta	*Chrysochromulinaceae*	1	0	1	0,00
Haptophyta	*Coccolithaceae*	2	1	1	50,00
Haptophyta	*Noelaerhabdaceae*	1	1	0	100,00
Haptophyta	*Halopappaceae*	1	1	0	100,00
Haptophyta	*Phaeocystaceae*	1	1	0	100,00
Haptophyta	*Pontosphaeraceae*	1	1	0	100,00
Haptophyta	*Rhabdosphaeraceae*	2	2	0	100,00
Charophyta	*Desmidiaceae*	1	1	0	100,00
Charophyta	*Closteriaceae*	1	1	0	100,00
Charophyta	*Zygnemataceae*	2	2	0	100,00
Cryptophyta	*Geminigeraceae*	1	1	0	100,00
Cryptophyta	*Katablepharidaceae*	1	0	1	0,00

Table S2. Number of species with and without 16S barcode for the families from transitional waters

**Phylum**	**Family**	**Total Number of species**	**Number of species without barcode**	**Number of species with barcode**	**Percentage of gap in the libraries**
Bacillariophyta	*Achnanthaceae*	1	0	1	0,00
Bacillariophyta	*Cocconeidaceae*	4	4	0	100,00
Bacillariophyta	*Fragilariaceae*	8	1	7	12,50

Bacillariophyta	*Chaetocerotaceae*	12	10	2	83,33
Bacillariophyta	*Climacospheniaceae*	1	1	0	100,00
Bacillariophyta	*Coscinodiscaceae*	3	2	1	66,67
Bacillariophyta	*Stephanodiscaceae*	3	1	2	33,33
Bacillariophyta	*Bacillariaceae*	12	6	6	50,00
Bacillariophyta	*Diploneidaceae*	3	3	0	100,00
Bacillariophyta	*Pinnulariaceae*	1	0	1	0,00
Bacillariophyta	*Stauroneidaceae*	1	1	0	100,00
Bacillariophyta	*Amphipleuraceae*	2	1	1	50,00
Bacillariophyta	*Naviculaceae*	3	2	1	66,67
Bacillariophyta	*Pleurosigmataceae*	3	3	0	100,00
Bacillariophyta	*Hemiaulaceae*	2	2	0	100,00
Bacillariophyta	*Leptocylindraceae*	2	1	1	50,00
Bacillariophyta	*Cymbellaceae*	1	1	0	100,00
Bacillariophyta	*Gomphonemataceae*	1	1	0	100,00
Bacillariophyta	*Rhizosoleniaceae*	7	5	2	71,43
Bacillariophyta	*Paraliaceae*	1	0	1	0,00
Bacillariophyta	*Striatellaceae*	3	3	0	100,00
Bacillariophyta	*Rhopalodiaceae*	1	1	0	100,00
Bacillariophyta	*Melosiraceae*	2	2	0	100,00
Bacillariophyta	*Catenulaceae*	3	0	3	0,00
Bacillariophyta	*Skeletonemaceae*	4	0	4	0,00
Bacillariophyta	*Thalassiosiraceae*	1	0	1	0,00
Bacillariophyta	*Surirellaceae*	1	1	0	100,00
Bacillariophyta	*Licmophoraceae*	3	3	0	100,00
Bacillariophyta	*Tabellariaceae*	1	0	1	0,00
Bacillariophyta	*Thalassionemataceae*	6	4	2	66,67
Bacillariophyta	*Mastogloiaceae*	1	1	0	100,00
Bacillariophyta	*Toxariaceae*	1	1	0	100,00
Cercozoa	*Ebriaceae*	1	1	0	100,00
Cyanobacteria	*Nostocaceae*	1	0	1	0,00
Cyanobacteria	*Chroococcaceae*	5	3	2	60,00
Cyanobacteria	*Microcystaceae*	4	1	3	25,00
Cyanobacteria	*Gomphosphaeriaceae*	1	0	1	0,00
Cyanobacteria	*Oscillatoriaceae*	5	1	4	20,00
Cyanobacteria	*Coelosphaeriaceae*	3	3	0	100,00
Cyanobacteria	*Merismopediaceae*	3	1	2	33,33
Cyanobacteria	*Spirulinaceae*	2	1	1	50,00
Chlorophyta	*Selenastraceae*	4	4	0	100,00
Chlorophyta	*Scenedesmaceae*	7	5	2	71,43
Chlorophyta	*Hydrodictyaceae*	3	2	1	66,67
Chlorophyta	*Trebouxiophyceae incertae sedis*	3	3	0	100,00
Chlorophyta	*Oocystaceae*	3	3	0	100,00
Chlorophyta	*Pyramimonadaceae*	1	0	1	0,00
Chlorophyta	*Prasiolaceae*	1	1	0	100,00

Chlorophyta	*Chlorodendraceae*	1	1	0	100,00
Chlorophyta	*Chlamydomonadaceae*	2	1	1	50,00
Euglenozoa	*Astasiaceae*	1	1	0	100,00
Euglenozoa	*Eutreptiaceae*	3	1	2	33,33
Euglenozoa	*Phacidae*	2	1	1	50,00
Euglenozoa	*Euglenidae*	1	1	0	100,00
Euglenozoa	*Euglenaceae*	3	1	2	33,33
Haptophyta	*Chrysochromulinaceae*	1	0	1	0,00
Haptophyta	*Coccolithaceae*	2	2	0	100,00
Haptophyta	*Noelaerhabdaceae*	1	1	0	100,00
Haptophyta	*Halopappaceae*	1	1	0	100,00
Haptophyta	*Phaeocystaceae*	1	1	0	100,00
Haptophyta	*Pontosphaeraceae*	1	1	0	100,00
Haptophyta	*Rhabdosphaeraceae*	2	1	1	50,00
Charophyta	*Desmidiaceae*	1	0	1	0,00
Charophyta	*Closteriaceae*	1	0	1	0,00
Charophyta	*Zygnemataceae*	2	1	1	50,00
Cryptophyta	*Geminigeraceae*	1	0	1	0,00
Cryptophyta	*Katablepharidaceae*	1	1	0	100,00

Table S3. Number of species with and without 18S barcode for the families from transitional waters

**Phylum**	**Family**	**Total Number of species**	**Number of species without barcode**	**Number of species with barcode**	**Percentage of gap in the libraries**
Bacillariophyta	*Achnanthaceae*	1	0	1	0,00
Bacillariophyta	*Cocconeidaceae*	4	2	2	50,00
Bacillariophyta	*Fragilariaceae*	8	1	7	12,50
Bacillariophyta	*Chaetocerotaceae*	12	2	10	16,67
Bacillariophyta	*Climacospheniaceae*	1	1	0	100,00
Bacillariophyta	*Coscinodiscaceae*	3	1	2	33,33
Bacillariophyta	*Stephanodiscaceae*	3	1	2	33,33
Bacillariophyta	*Bacillariaceae*	12	1	11	8,33
Bacillariophyta	*Diploneidaceae*	3	2	1	66,67
Bacillariophyta	*Pinnulariaceae*	1	0	1	0,00
Bacillariophyta	*Stauroneidaceae*	1	0	1	0,00
Bacillariophyta	*Amphipleuraceae*	2	1	1	50,00
Bacillariophyta	*Naviculaceae*	3	2	1	66,67
Bacillariophyta	*Pleurosigmataceae*	3	2	1	66,67
Bacillariophyta	*Hemiaulaceae*	2	1	1	50,00
Bacillariophyta	*Leptocylindraceae*	2	0	2	0,00
Bacillariophyta	*Cymbellaceae*	1	0	1	0,00
Bacillariophyta	*Gomphonemataceae*	1	0	1	0,00
Bacillariophyta	*Rhizosoleniaceae*	7	2	5	28,57
Bacillariophyta	*Paraliaceae*	1	0	1	0,00
Bacillariophyta	*Striatellaceae*	3	0	3	0,00
Bacillariophyta	*Rhopalodiaceae*	1	0	1	0,00

Bacillariophyta	*Melosiraceae*	2	0	2	0,00
Bacillariophyta	*Catenulaceae*	3	0	3	0,00
Bacillariophyta	*Skeletonemaceae*	4	0	4	0,00
Bacillariophyta	*Thalassiosiraceae*	1	0	1	0,00
Bacillariophyta	*Surirellaceae*	1	1	0	100,00
Bacillariophyta	*Licmophoraceae*	3	0	3	0,00
Bacillariophyta	*Tabellariaceae*	1	1	0	100,00
Bacillariophyta	*Thalassionemataceae*	6	2	4	33,33
Bacillariophyta	*Mastogloiaceae*	1	1	0	100,00
Bacillariophyta	*Toxariaceae*	1	0	1	0,00
Ochrophyta	*Chrysococcaceae*	1	1	0	100,00
Ochrophyta	*Dinobryaceae*	4	2	2	50,00
Ochrophyta	*Dictyochacea*	2	1	1	50,00
Ochrophyta	*Pleurochloridaceae*	2	2	0	100,00
Ochrophyta	*Pedinellaceae*	1	1	0	100,00
Myzozoa	*Gymnodiniaceae*	6	3	3	50,00
Myzozoa	*Polykrikaceae*	2	1	1	50,00
Myzozoa	*Gyrodiniaceae*	3	1	2	33,33
Myzozoa	*Amphidiniaceae*	4	2	2	50,00
Myzozoa	*Dinophysaceae*	1	1	0	100,00
Myzozoa	*Peridiniaceae*	4	1	3	25,00
Myzozoa	*Oxytoxaceae*	2	2	0	100,00
Myzozoa	*Heterocapsaceae*	4	1	3	25,00
Myzozoa	*Protoperidiniaceae*	13	10	3	76,92
Myzozoa	*Gonyaulacaceae*	6	2	4	33,33
Myzozoa	*Ceratiaceae*	2	0	2	0,00
Myzozoa	*Ostreopsidaceae*	3	0	3	0,00
Myzozoa	*Tovelliaceae*	1	1	0	100,00
Myzozoa	*Thoracosphaeraceae*	1	1	0	100,00
Myzozoa	*Oxyrrhinaceae*	1	0	1	0,00
Myzozoa	*Prorocentraceae*	14	8	6	57,14
Cercozoa	*Ebriaceae*	1	0	1	0,00
Chlorophyta	*Selenastraceae*	4	3	1	75,00
Chlorophyta	*Scenedesmaceae*	7	0	7	0,00
Chlorophyta	*Hydrodictyaceae*	3	1	2	33,33
Chlorophyta	*Trebouxiophyceae incertae sedis*	3	3	0	100,00
Chlorophyta	*Oocystaceae*	3	1	2	33,33
Chlorophyta	*Pyramimonadaceae*	1	0	1	0,00
Chlorophyta	*Prasiolaceae*	1	1	0	100,00
Chlorophyta	*Chlorodendraceae*	1	0	1	0,00
Chlorophyta	*Chlamydomonadaceae*	2	0	2	0,00
Euglenozoa	*Astasiaceae*	1	0	1	0,00
Euglenozoa	*Eutreptiaceae*	3	1	2	33,33
Euglenozoa	*Phacidae*	2	0	2	0,00
Euglenozoa	*Euglenidae*	1	0	1	0,00

Euglenozoa	*Euglenaceae*	3	0	3	0,00
Haptophyta	*Chrysochromulinaceae*	1	0	1	0,00
Haptophyta	*Coccolithaceae*	2	2	0	100,00
Haptophyta	*Noelaerhabdaceae*	1	1	0	100,00
Haptophyta	*Halopappaceae*	1	1	0	100,00
Haptophyta	*Phaeocystaceae*	1	0	1	0,00
Haptophyta	*Pontosphaeraceae*	1	1	0	100,00
Haptophyta	*Rhabdosphaeraceae*	2	1	1	50,00
Charophyta	*Desmidiaceae*	1	0	1	0,00
Charophyta	*Closteriaceae*	1	0	1	0,00
Charophyta	*Zygnemataceae*	2	0	2	0,00
Cryptophyta	*Geminigeraceae*	1	1	0	100,00
Cryptophyta	*Katablepharidaceae*	1	0	1	0,00

Table S4. Number of species with and without COI barcode for the families from lakes

**Phylum**	**Family**	**Total Number of species**	**Number of species without barcode**	**Number of species with barcode**	**Percentage of gap in the libraries**
Bacillariophyta	*Achnanthaceae*	1	1	0	100,00
Bacillariophyta	*Cocconeidaceae*	2	2	0	100,00
Bacillariophyta	*Catenulaceae*	2	2	0	100,00
Bacillariophyta	*Fragilariaceae*	8	8	0	100,00
Bacillariophyta	*Stephanodiscaceae*	6	5	1	83,33
Bacillariophyta	*Cymbellaceae*	2	2	0	100,00
Bacillariophyta	*Striatellaceae*	1	1	0	100,00
Bacillariophyta	*Bacillariaceae*	7	4	3	57,14
Bacillariophyta	*Pinnulariaceae*	1	0	1	0,00
Bacillariophyta	*Pleurosigmataceae*	2	2	0	100,00
Bacillariophyta	*Stauroneidaceae*	2	2	0	100,00
Bacillariophyta	*Naviculaceae*	3	1	2	33,33
Bacillariophyta	*Thalassiosiraceae*	1	1	0	100,00
Bacillariophyta	*Thalassionemataceae*	1	0	1	0,00
Bacillariophyta	*Ulnariaceae*	1	1	0	100,00
Bacillariophyta	*Aulacoseiraceae*	1	1	0	100,00
Bacillariophyta	*Melosiraceae*	1	1	0	100,00
Ochrophyta	*Dinobryaceae*	2	0	2	0,00
Ocrhophyta	*Mallomonadaceae*	1	0	1	0,00
Ochrophyta	*Tribonemetaceae*	1	1	0	100,00
Myzozoa	*Amphidiniaceae*	1	1	0	100,00
Myzozoa	*Ceratiaceae*	1	0	1	0,00
Myzozoa	*Phytodiniaceae*	1	1	0	100,00
Myzozoa	*Gymnodiniaceae*	1	0	1	0,00
Myzozoa	*Peridiniaceae*	3	2	1	66,67
Cyanobacteria	*Nostocaceae*	2	1	1	50,00
Cyanobacteria	*Merismopediaceae*	4	3	1	75,00
Cyanobacteria	*Coelosphaeriaceae*	2	2	0	100,00

Cyanobacteria	*Microcystaceae*	3	2	1	66,67
Cyanobacteria	*Chroococcaceae*	5	5	0	100,00
Cyanobacteria	*Gomphosphaeriaceae*	1	1	0	100,00
Cyanobacteria	*Oscillatoriaceae*	2	2	0	100,00
Cyanobacteria	*Microcoleaceae*	1	1	0	100,00
Charophyta	*Closteriaceae*	8	8	0	100,00
Charophyta	*Desmidiaceae*	6	6	0	100,00
Charophyta	*Elakatotrichaceae*	3	3	0	100,00
Cryptophyta	*Cryptomonadaceae*	1	0	1	0,00
Cryptophyta	*Pyrenomonadaceae*	1	0	1	0,00
Euglenozoa	*Euglenaceae*	2	2	0	100,00
Euglenozoa	*Phacidae*	3	3	0	100,00
Euglenozoa	*Euglenidae*	1	1	0	100,00

Table S5. Number of species with and without 16S barcode for the families from lakes

**Phylum**	**Family**	**Total Number of species**	**Number of species without barcode**	**Number of species with barcode**	**Percentage of gap in the libraries**
Bacillariophyta	*Achnanthaceae*	1	0	1	0,00
Bacillariophyta	*Cocconeidaceae*	2	2	0	100,00
Bacillariophyta	*Catenulaceae*	2	0	2	0,00
Bacillariophyta	*Fragilariaceae*	8	1	7	12,50
Bacillariophyta	*Stephanodiscaceae*	6	3	3	50,00
Bacillariophyta	*Cymbellaceae*	2	2	0	100,00
Bacillariophyta	*Striatellaceae*	1	1	0	100,00
Bacillariophyta	*Bacillariaceae*	7	5	2	71,43
Bacillariophyta	*Pinnulariaceae*	1	0	1	0,00
Bacillariophyta	*Pleurosigmataceae*	2	2	0	100,00
Bacillariophyta	*Stauroneidaceae*	2	2	0	100,00
Bacillariophyta	*Naviculaceae*	3	1	2	33,33
Bacillariophyta	*Thalassiosiraceae*	1	0	1	0,00
Bacillariophyta	*Thalassionemataceae*	1	0	1	0,00
Bacillariophyta	*Ulnariaceae*	1	1	0	100,00
Bacillariophyta	*Aulacoseiraceae*	1	0	1	0,00
Bacillariophyta	*Melosiraceae*	1	1	0	100,00
Cyanobacteria	*Nostocaceae*	2	0	2	0,00
Cyanobacteria	*Merismopediaceae*	4	2	2	50,00
Cyanobacteria	*Coelosphaeriaceae*	2	0	2	0,00
Cyanobacteria	*Microcystaceae*	3	1	2	33,33
Cyanobacteria	*Chroococcaceae*	5	2	3	40,00
Cyanobacteria	*Gomphosphaeriaceae*	1	1	0	100,00
Cyanobacteria	*Oscillatoriaceae*	2	0	2	0,00
Cyanobacteria	*Microcoleaceae*	1	0	1	0,00
Chlorophyta	*Chlamydomonadaceae*	3	3	0	100,00
Chlorophyta	*Chlorococcaceae*	1	1	0	100,00

Chlorophyta	*Palmellaceae*	1	1	0	100,00
Chlorophyta	*Sphaerocystidaeae*	1	1	0	100,00
Chlorophyta	*Oocystaceae*	6	5	1	83,33
Chlorophyta	*Chlorellaceae*	1	1	0	100,00
Chlorophyta	*Hydrodictyaceae*	9	7	2	77,78
Chlorophyta	*Chlorodendraceae*	1	1	0	100,00
Chlorophyta	*Trebouxiophyceae incertae sedis*	5	5	0	100,00
Chlorophyta	*Radiococcaceae*	4	4	0	100,00
Chlorophyta	*Schizochlamydaceae*	1	1	0	100,00
Chlorophyta	*Characiaceae*	1	1	0	100,00
Chlorophyta	*Treubariaceae*	2	2	0	100,00
Chlorophyta	*Schroederiaceae*	2	1	1	50,00
Chlorophyta	*Scenedesmaceae*	21	18	3	85,71
Chlorophyta	*Selenastraceae*	11	7	4	63,64
Chlorophyta	*Neochloridaceae*	1	1	0	100,00
Charophyta	*Closteriaceae*	8	6	2	75,00
Charophyta	*Desmidiaceae*	6	3	3	50,00
Charophyta	*Elakatotrichaceae*	3	3	0	100,00
Cryptophyta	*Cryptomonadaceae*	1	0	1	0,00
Cryptophyta	*Pyrenomonadaceae*	1	0	1	0,00
Euglenozoa	*Euglenaceae*	2	1	1	50,00
Euglenozoa	*Phacidae*	3	1	2	33,33
Euglenozoa	*Euglenidae*	1	1	0	100,00

Table S6. Number of species with and without 18S barcode for the families from lakes

**Phylum**	**Family**	**Total Number of species**	**Number of species without barcode**	**Number of species with barcode**	**Percentage of gap in the libraries**
Bacillariophyta	*Achnanthaceae*	1	0	1	0,00
Bacillariophyta	*Cocconeidaceae*	2	1	1	50,00
Bacillariophyta	*Catenulaceae*	2	0	2	0,00
Bacillariophyta	*Fragilariaceae*	8	1	7	12,50
Bacillariophyta	*Stephanodiscaceae*	6	3	3	50,00
Bacillariophyta	*Cymbellaceae*	2	0	2	0,00
Bacillariophyta	*Striatellaceae*	1	0	1	0,00
Bacillariophyta	*Bacillariaceae*	7	0	7	0,00
Bacillariophyta	*Pinnulariaceae*	1	0	1	0,00
Bacillariophyta	*Pleurosigmataceae*	2	1	1	50,00
Bacillariophyta	*Stauroneidaceae*	2	0	2	0,00
Bacillariophyta	*Naviculaceae*	3	1	2	33,33
Bacillariophyta	*Thalassiosiraceae*	1	0	1	0,00
Bacillariophyta	*Thalassionemataceae*	1	0	1	0,00
Bacillariophyta	*Ulnariaceae*	1	0	1	0,00
Bacillariophyta	*Aulacoseiraceae*	1	0	1	0,00
Bacillariophyta	*Melosiraceae*	1	0	1	0,00

Ochrophyta	*Dinobryaceae*	2	0	2	0,00
Ochrophyta	*Mallomonadaceae*	1	0	1	0,00
Ochrophyta	*Tribonemetaceae*	1	0	1	0,00
Myzozoa	*Amphidiniaceae*	1	1	0	100,00
Myzozoa	*Ceratiaceae*	1	0	1	0,00
Myzozoa	*Phytodiniaceae*	1	1	0	100,00
Myzozoa	*Gymnodiniaceae*	1	0	1	0,00
Myzozoa	*Peridiniaceae*	3	2	1	66,67
Chlorophyta	*Chlamydomonadaceae*	3	1	2	33,33
Chlorophyta	*Chlorococcaceae*	1	1	0	100,00
Chlorophyta	*Palmellaceae*	1	1	0	100,00
Chlorophyta	*Sphaerocystidaeae*	1	0	1	0,00
Chlorophyta	*Oocystaceae*	6	2	4	33,33
Chlorophyta	*Chlorellaceae*	1	0	1	0,00
Chlorophyta	*Hydrodictyaceae*	9	2	7	22,22
Chlorophyta	*Chlorodendraceae*	1	0	1	0,00
Chlorophyta	*Trebouxiophyceae incertae sedis*	5	5	0	100,00
Chlorophyta	*Radiococcaceae*	4	1	3	25,00
Chlorophyta	*Schizochlamydaceae*	1	1	0	100,00
Chlorophyta	*Characiaceae*	1	0	1	0,00
Chlorophyta	*Treubariaceae*	2	0	2	0,00
Chlorophyta	*Schroederiaceae*	2	1	1	50,00
Chlorophyta	*Scenedesmaceae*	21	10	11	47,62
Chlorophyta	*Selenastraceae*	11	3	8	27,27
Chlorophyta	*Neochloridaceae*	1	0	1	0,00
Charophyta	*Closteriaceae*	8	5	3	62,50
Charophyta	*Desmidiaceae*	6	0	6	0,00
Charophyta	*Elakatotrichaceae*	3	3	0	100,00
Cryptophyta	*Cryptomonadaceae*	1	0	1	0,00
Cryptophyta	*Pyrenomonadaceae*	1	0	1	0,00
Euglenozoa	*Euglenaceae*	2	1	1	50,00
Euglenozoa	*Phacidae*	3	0	3	0,00
Euglenozoa	*Euglenidae*	1	1	0	100,00

Table S7. Number of species with and without COI barcode for the families from marine coastal waters

**Phylum**	**Family**	**Total Number of species**	**Number of species without barcode**	**Number of species with barcode**	**Percentage of gap in the libraries**
Bacillariophyta	*Achnanthaceae*	3	3	0	100,00
Bacillariophyta	*Cocconeidaceae*	4	4	0	100,00
Bacillariophyta	*Fragilariaceae*	12	9	3	75,00
Bacillariophyta	*Hemiaulaceae*	6	6	0	100,00
Bacillariophyta	*Licmophoraceae*	6	6	0	100,00
Bacillariophyta	*Leptocylindraceae*	4	3	1	75,00
Bacillariophyta	*Chaetocerotaceae*	32	17	15	53,13

Bacillariophyta	*Attheyaceae*	1	1	0	100,00
Bacillariophyta	*Stephanodiscaceae*	2	0	2	0,00
Bacillariophyta	*Biddulphiaceae*	2	2	0	100,00
Bacillariophyta	*Cymbellaceae*	1	1	0	100,00
Bacillariophyta	*Gomphonemataceae*	1	1	0	100,00
Bacillariophyta	*Climacospheniaceae*	2	2	0	100,00
Bacillariophyta	*Bacillariaceae*	21	12	9	57,14
Bacillariophyta	*Pleurosigmataceae*	5	5	0	100,00
Bacillariophyta	*Naviculaceae*	10	7	3	70,00
Bacillariophyta	*Diploneidaceae*	3	3	0	100,00
Bacillariophyta	*Pinnulariaceae*	1	0	1	0,00
Bacillariophyta	*Amphipleuraceae*	1	1	0	100,00
Bacillariophyta	*Thalassionemataceae*	8	7	1	87,50
Bacillariophyta	*Melosiraceae*	1	1	0	100,00
Bacillariophyta	*Skeletonemaceae*	6	3	3	50,00
Bacillariophyta	*Thalassiosiraceae*	6	3	3	50,00
Bacillariophyta	*Lauderiaceae*	2	2	0	100,00
Bacillariophyta	*Striatellaceae*	2	2	0	100,00
Bacillariophyta	*Entomoneidaceae*	2	2	0	100,00
Bacillariophyta	*Surirellaceae*	1	1	0	100,00
Bacillariophyta	*Paraliaceae*	1	1	0	100,00
Bacillariophyta	*Lithodesmiaceae*	1	0	1	0,00
Bacillariophyta	*Coscinodiscaceae*	4	2	2	50,00
Bacillariophyta	*Triceratiaceae*	1	1	0	100,00
Bacillariophyta	*Toxariaceae*	1	1	0	100,00
Bacillariophyta	*Tabellariaceae*	1	1	0	100,00
Bacillariophyta	*Rhizosoleniaceae*	17	16	1	94,12
Bacillariophyta	*Catenulaceae*	3	3	0	100,00
Bacillariophyta	*Asterolampraceae*	1	1	0	100,00
Bacillariophyta	*Pleurochloridaceae*	2	2	0	100,00
Ochrophyta	*Chattonellaceae*	1	1	0	100,00
Ochrophyta	*Pedinellaceae*	1	1	0	100,00
Ochrophyta	*Dinobryaceae*	2	1	1	50,00
Ochrophyta	*Dictyochacea*	6	5	1	83,33
Myzozoa	*Gymnodiniaceae*	12	6	6	50,00
Myzozoa	*Brachidiniaceae*	1	1	0	100,00
Myzozoa	*Warnowiaceae*	1	1	0	100,00
Myzozoa	*Gyrodiniaceae*	3	3	0	100,00
Myzozoa	*Peridiniaceae*	6	2	4	33,33
Myzozoa	*Heterocapsaceae*	5	5	0	100,00
Myzozoa	*Oxytoxaceae*	13	13	0	100,00
Myzozoa	*Podolampadaceae*	3	3	0	100,00
Myzozoa	*Protoperidiniaceae*	20	19	1	95,00
Myzozoa	*Dinophysaceae*	12	5	7	41,67
Myzozoa	*Gonyaulacaceae*	8	3	5	37,50
Myzozoa	*Ostreopsidaceae*	6	0	6	0,00

Myzozoa	*Ceratiaceae*	11	11	0	100,00
Myzozoa	*Ceratocoryaceae*	1	0	1	0,00
Myzozoa	*Thoracosphaeraceae*	1	1	0	100,00
Myzozoa	*Prorocentraceae*	17	9	8	52,94
Myzozoa	*Tovelliaceae*	2	2	0	100,00
Myzozoa	*Kofoidiniaceae*	3	3	0	100,00
Myzozoa	*Noctilucaceae*	1	1	0	100,00
Myzozoa	*Pyrocystaceae*	1	1	0	100,00
Myzozoa	*Amphidiniaceae*	5	5	0	100,00
Haptophyta	*Rhabdoshpaeraceae*	10	10	0	100,00
Haptophyta	*Syracosphaeraceae*	3	3	0	100,00
Haptophyta	*Coccolithaceae*	1	0	1	0,00
Haptophyta	*Calyptrosphaeraceae*	6	6	0	100,00
Haptophyta	*Halopappaceae*	1	1	0	100,00
Haptophyta	*Noelaerhabdaceae*	1	0	1	0,00
Haptophyta	*Phaeocystaceae*	1	1	0	100,00
Haptophyta	*Pontosphaeraceae*	1	1	0	100,00
Haptophyta	*Prymnesiaceae*	1	1	0	100,00
Haptophyta	*Chrysochromulinaceae*	2	0	2	0,00
Haptophyta	*Ceratolithaceae*	2	2	0	100,00
Charophyta	*Closteriaceae*	1	1	0	100,00
Charophyta	*Desmidiaceae*	1	1	0	100,00
Charophyta	*Elakatotrichaceae*	1	1	0	100,00
Cercozoa	*Ebriaceae*	2	2	0	100,00
Euglenozoa	*Euglenaceae*	1	1	0	100,00
Euglenozoa	*Eutreptiaceae*	3	3	0	100,00
Cyanobacteria	*Coelosphaeriaceae*	1	1	0	100,00
Cyanobacteria	*Spirulinaceae*	1	1	0	100,00
Cyanobacteria	*Oscillatoriaceae*	3	3	0	100,00
Cryptophyta	*Hilleaceae*	1	1	0	100,00
Cryptophyta	*Katablepharidaceae*	1	1	0	100,00
Cryptophyta	*Geminigeraceae*	3	3	0	100,00

Table S8. Number of species with and without 16S barcode for the families from marine coastal waters

**Phylum**	**Family**	**Total Number of species**	**Number of species without barcode**	**Number of species with barcode**	**Percentage of gap in the libraries**
Bacillariophyta	*Achnanthaceae*	3	0	3	0,00
Bacillariophyta	*Cocconeidaceae*	4	4	0	100,00
Bacillariophyta	*Fragilariaceae*	12	4	8	33,33
Bacillariophyta	*Hemiaulaceae*	6	6	0	100,00
Bacillariophyta	*Leptocylindraceae*	4	3	1	75,00
Bacillariophyta	*Licmophoraceae*	6	6	0	100,00
Bacillariophyta	*Chaetocerotaceae*	32	27	5	84,38
Bacillariophyta	*Attheyaceae*	1	1	0	100,00
Bacillariophyta	*Stephanodiscaceae*	2	0	2	0,00

Bacillariophyta	*Biddulphiaceae*	2	2	0	100,00
Bacillariophyta	*Cymbellaceae*	1	1	0	100,00
Bacillariophyta	*Gomphonemataceae*	1	1	0	100,00
Bacillariophyta	*Climacospheniaceae*	2	2	0	100,00
Bacillariophyta	*Bacillariaceae*	21	8	13	38,10
Bacillariophyta	*Pleurosigmataceae*	5	5	0	100,00
Bacillariophyta	*Naviculaceae*	10	6	4	60,00
Bacillariophyta	*Diploneidaceae*	3	3	0	100,00
Bacillariophyta	*Pinnulariaceae*	1	0	1	0,00
Bacillariophyta	*Amphipleuraceae*	1	1	0	100,00
Bacillariophyta	*Thalassionemataceae*	8	5	3	62,50
Bacillariophyta	*Melosiraceae*	1	1	0	100,00
Bacillariophyta	*Skeletonemaceae*	6	1	5	16,67
Bacillariophyta	*Thalassiosiraceae*	6	0	6	0,00
Bacillariophyta	*Lauderiaceae*	2	1	1	50,00
Bacillariophyta	*Striatellaceae*	2	2	0	100,00
Bacillariophyta	*Entomoneidaceae*	2	2	0	100,00
Bacillariophyta	*Surirellaceae*	1	1	0	100,00
Bacillariophyta	*Paraliaceae*	1	0	1	0,00
Bacillariophyta	*Lithodesmiaceae*	1	0	1	0,00
Bacillariophyta	*Coscinodiscaceae*	4	1	3	25,00
Bacillariophyta	*Triceratiaceae*	1	0	1	0,00
Bacillariophyta	*Toxariaceae*	1	1	0	100,00
Bacillariophyta	*Tabellariaceae*	1	0	1	0,00
Bacillariophyta	*Rhizosoleniaceae*	17	14	3	82,35
Bacillariophyta	*Catenulaceae*	3	0	3	0,00
Bacillariophyta	*Asterolampraceae*	1	1	0	100,00
Bacillariophyta	*Pleurochloridaceae*	2	2	0	100,00
Haptophyta	*Rhabdoshpaeraceae*	10	10	0	100,00
Haptophyta	*Syracosphaeraceae*	3	2	1	66,67
Haptophyta	*Coccolithaceae*	1	1	0	100,00
Haptophyta	*Calyptrosphaeraceae*	6	6	0	100,00
Haptophyta	*Halopappaceae*	1	1	0	100,00
Haptophyta	*Noelaerhabdaceae*	1	1	0	100,00
Haptophyta	*Phaeocystaceae*	1	1	0	100,00
Haptophyta	*Pontosphaeraceae*	1	1	0	100,00
Haptophyta	*Prymnesiaceae*	1	0	1	0,00
Haptophyta	*Chrysochromulinaceae*	2	0	2	0,00
Haptophyta	*Ceratolithaceae*	2	2	0	100,00
Chlorophyta	*Selenastraceae*	5	4	1	80,00
Chlorophyta	*Scenedesmaceae*	3	1	2	33,33
Chlorophyta	*Hydrodictyaceae*	1	1	0	100,00
Chlorophyta	*Chlamydomonadaceae*	1	1	0	100,00
Chlorophyta	*Chlorodendraceae*	1	1	0	100,00
Chlorophyta	*Chlorellaceae*	1	0	1	0,00
Chlorophyta	*Hydrodictyaceae*	1	0	1	0,00

Chlorophyta	*Pyramimonadaceae*	1	0	1	0,00
Chlorophyta	*Trebouxiophyceae incertae sedis*	2	2	0	100,00
Charophyta	*Closteriaceae*	1	0	1	0,00
Charophyta	*Desmidiaceae*	1	1	0	100,00
Charophyta	*Elakatotrichaceae*	1	1	0	100,00
Cercozoa	*Ebriaceae*	2	2	0	100,00
Euglenozoa	*Euglenaceae*	1	0	1	0,00
Euglenozoa	*Eutreptiaceae*	3	1	2	33,33
Cyanobacteria	*Coelosphaeriaceae*	1	1	0	100,00
Cyanobacteria	*Spirulinaceae*	1	0	1	0,00
Cyanobacteria	*Oscillatoriaceae*	3	0	3	0,00
Cryptophyta	*Hilleaceae*	1	1	0	100,00
Cryptophyta	*Katablepharidaceae*	1	1	0	100,00
Cryptophyta	*Geminigeraceae*	3	0	3	0,00

Table S9. Number of species with and without 18S barcode for the families from marine coastal waters

**Phylum**	**Family**	**Total Number of species**	**Number of species without barcode**	**Number of species with barcode**	**Percentage of gap in the libraries**
Bacillariophyta	*Achnanthaceae*	3	0	3	0,00
Bacillariophyta	*Cocconeidaceae*	4	1	3	25,00
Bacillariophyta	*Fragilariaceae*	12	3	9	25,00
Bacillariophyta	*Hemiaulaceae*	6	2	4	33,33
Bacillariophyta	*Leptocylindraceae*	4	1	3	25,00
Bacillariophyta	*Licmophoraceae*	6	1	5	16,67
Bacillariophyta	*Chaetocerotaceae*	32	8	24	25,00
Bacillariophyta	*Attheyaceae*	1	0	1	0,00
Bacillariophyta	*Stephanodiscaceae*	2	0	2	0,00
Bacillariophyta	*Biddulphiaceae*	2	1	1	50,00
Bacillariophyta	*Cymbellaceae*	1	0	1	0,00
Bacillariophyta	*Gomphonemataceae*	1	0	1	0,00
Bacillariophyta	*Climacospheniaceae*	2	0	2	0,00
Bacillariophyta	*Bacillariaceae*	21	3	18	14,29
Bacillariophyta	*Pleurosigmataceae*	5	3	2	60,00
Bacillariophyta	*Naviculaceae*	10	5	5	50,00
Bacillariophyta	*Diploneidaceae*	3	1	2	33,33
Bacillariophyta	*Pinnulariaceae*	1	0	1	0,00
Bacillariophyta	*Amphipleuraceae*	1	1	0	100,00
Bacillariophyta	*Thalassionemataceae*	8	3	5	37,50
Bacillariophyta	*Melosiraceae*	1	0	1	0,00
Bacillariophyta	*Skeletonemaceae*	6	0	6	0,00
Bacillariophyta	*Thalassiosiraceae*	6	1	5	16,67
Bacillariophyta	*Lauderiaceae*	2	1	1	50,00
Bacillariophyta	*Striatellaceae*	2	0	2	0,00
Bacillariophyta	*Entomoneidaceae*	2	0	2	0,00

Bacillariophyta	*Surirellaceae*	1	0	1	0,00
Bacillariophyta	*Paraliaceae*	1	0	1	0,00
Bacillariophyta	*Lithodesmiaceae*	1	0	1	0,00
Bacillariophyta	*Coscinodiscaceae*	4	1	3	25,00
Bacillariophyta	*Triceratiaceae*	1	0	1	0,00
Bacillariophyta	*Toxariaceae*	1	0	1	0,00
Bacillariophyta	*Tabellariaceae*	1	1	0	100,00
Bacillariophyta	*Rhizosoleniaceae*	17	4	13	23,53
Bacillariophyta	*Catenulaceae*	3	0	3	0,00
Bacillariophyta	*Asterolampraceae*	1	0	1	0,00
Bacillariophyta	*Pleurochloridaceae*	2	2	0	100,00
Ochrophyta	*Pedinellaceae*	1	1	0	100,00
Ochrophyta	*Dinobryaceae*	2	1	1	50
Ochrophyta	*Dictyochacea*	6	4	2	66,67
Ochrophyta	*Chattonellaceae*	1	1	0	100
Myzozoa	*Gymnodiniaceae*	12	3	9	25,00
Myzozoa	*Brachidiniaceae*	1	0	1	0,00
Myzozoa	*Warnowiaceae*	1	0	1	0,00
Myzozoa	*Gyrodiniaceae*	3	0	3	0,00
Myzozoa	*Peridiniaceae*	6	3	3	50,00
Myzozoa	*Heterocapsaceae*	5	1	4	20,00
Myzozoa	*Oxytoxaceae*	13	13	0	100,00
Myzozoa	*Podolampadaceae*	3	1	2	33,33
Myzozoa	*Protoperidiniaceae*	20	16	4	80,00
Myzozoa	*Dinophysaceae*	12	3	9	25,00
Myzozoa	*Gonyaulacaceae*	8	3	5	37,50
Myzozoa	*Ostreopsidaceae*	6	0	6	0,00
Myzozoa	*Ceratiaceae*	11	9	2	81,82
Myzozoa	*Ceratocoryaceae*	1	0	1	0,00
Myzozoa	*Thoracosphaeraceae*	1	0	1	0,00
Myzozoa	*Prorocentraceae*	17	9	8	52,94
Myzozoa	*Tovelliaceae*	2	2	0	100,00
Myzozoa	*Kofoidiniaceae*	3	1	2	33,33
Myzozoa	*Noctilucaceae*	1	0	1	0,00
Myzozoa	*Pyrocystaceae*	1	0	1	0,00
Myzozoa	*Amphidiniaceae*	5	3	2	60,00
Haptophyta	*Rhabdoshpaeraceae*	10	10	0	100,00
Haptophyta	*Syracosphaeraceae*	3	2	1	66,67
Haptophyta	*Coccolithaceae*	1	1	0	100,00
Haptophyta	*Calyptrosphaeraceae*	6	4	2	66,67
Haptophyta	*Halopappaceae*	1	1	0	100,00
Haptophyta	*Noelaerhabdaceae*	1	1	0	100,00
Haptophyta	*Phaeocystaceae*	1	0	1	0,00
Haptophyta	*Pontosphaeraceae*	1	1	0	100,00
Haptophyta	*Prymnesiaceae*	1	0	1	0,00
Haptophyta	*Chrysochromulinaceae*	2	0	2	0,00

Haptophyta	*Ceratolithaceae*	2	2	0	100,00
Chlorophyta	*Selenastraceae*	5	1	4	20,00
Chlorophyta	*Scenedesmaceae*	3	0	3	0,00
Chlorophyta	*Hydrodictyaceae*	1	0	1	0,00
Chlorophyta	*Chlamydomonadaceae*	1	0	1	0,00
Chlorophyta	*Chlorodendraceae*	1	0	1	0,00
Chlorophyta	*Chlorellaceae*	1	0	1	0,00
Chlorophyta	*Hydrodictyaceae*	1	0	1	0,00
Chlorophyta	*Pyramimonadaceae*	1	0	1	0,00
Chlorophyta	*Trebouxiophyceae incertae sedis*	2	2	0	100,00
Charophyta	*Closteriaceae*	1	0	1	0,00
Charophyta	*Desmidiaceae*	1	1	0	100,00
Charophyta	*Elakatotrichaceae*	1	1	0	100,00
Cercozoa	*Ebriaceae*	2	1	1	50,00
Euglenozoa	*Euglenaceae*	1	0	1	0,00
Euglenozoa	*Eutreptiaceae*	3	1	2	33,33
Cryptophyta	*Hilleaceae*	1	1	0	100,00
Cryptophyta	*Katablepharidaceae*	1	1	0	100,00
Cryptophyta	*Geminigeraceae*	3	2	1	66,67
